# The histological development of the fetal human inferior colliculus during the second trimester

**DOI:** 10.3389/fnana.2024.1502778

**Published:** 2025-01-06

**Authors:** Reetuparna Nanda, Mihail Bota, Jaikishan Jayakumar, Suresh S, S. Lata, E. Harish Kumar, Chitra Srinivasan, Sudha Vasudevan, Kumutha Jayaraman, Mohanasankar Sivaprakasam, Richa Verma

**Affiliations:** ^1^Sudha Gopalakrishnan Brain Centre, Indian Institute of Technology Madras, Chennai, India; ^2^Center for Computational Brain Research, Indian Institute of Technology Madras, Chennai, India; ^3^Mediscan Systems, Chennai, India; ^4^Department of Pathology, Saveetha Medical College, Thandalam, Tamil Nadu, Chennai, India; ^5^Department of Neonatology, Saveetha Medical College, Thandalam, Tamil Nadu, Chennai, India; ^6^Healthcare Technology Innovation Centre, Indian Institute of Technology Madras, Chennai, India; ^7^Department of Electrical Engineering, Indian Institute of Technology, Madras, Chennai, India

**Keywords:** inferior colliculus, central nucleus, dorsal cortex, external cortex, cytoarchitecture, GFAP, calretinin

## Abstract

The inferior colliculus (IC) is an important midbrain station of the auditory pathway, as well as an important hub of multisensory integration. The adult mammalian IC can be subdivided into three nuclei, with distinct cyto- and myeloarchitectonical profiles and distinct calcium binding proteins expression patterns. Despite several studies about its structural and functional development, the knowledge about the human fetal IC is rather limited. In this paper we first systematically describe the histological development of the human fetal IC and its subparts in five stages of the second trimester of pregnancy: 15 gestation weeks (GW), 18 GW, 21 GW, 24 GW, and 27 GW. We 3D reconstruct and calculate the volumetric growth of IC from one stage to another, which increases from 12.85 mm^3^ at 15 GW to 34.27 mm^3^ at 27 GW in the left hemisphere. The volumetric changes in the IC were further evaluated at the cellular level using serial Nissl-stained sections, as well as glial fibrillary acidic proteins (GFAP) and calretinin immunohistochemistry. We identify stellate-like and round neurons in the central nucleus of the IC (CNIC) at 24 GW and 27 GW, comparable to the adult human IC. Novel in this study, we investigate the differential calretinin expression patterns in the IC subparts, from 15 GW to 27 GW. CR labeling is identified mainly in the cortical IC rather than in the central nucleus. Furthermore, using GFAP, we describe the radial glial fibers patterns in IC, which are dominant at 18 GW and gradually taper off at later developmental stages. Finally, we describe the development of astroglia in each of the five developmental stages. All these results indicate that the human fetal IC development and cellular maturation occur in two major stages during the second trimester.

## 1 Introduction

The inferior colliculus (IC) has been classically described as two elevated structures, comprising the caudal (posterior) part of the tectum (roof of the mesencephalon; Olszewski and Baxter, 1982), and located rostrally (anteriorly) from the entry of the trochlear nerve (Driscoll and Tadi, 2022). The adult human IC can be subdivided into two parts, the central nucleus (CNIC) and the cortical part. Based on Nissl and Golgi staining as well as immunoreactivity (IR) of specific calcium-binding proteins, the latter is further divided into the external cortex (ECIC) and the dorsal cortex (DCIC; Geniec and Morest, 1971; Mansour et al., 2019; Olszewski and Baxter, 1982; Tardif et al., 2003). The developmental cytoarchitectural patterns of parvalbumin (PV) and calbindin (CB) in the fetal IC have been already described (Sharma et al., 2009a). However, calretinin (CR) has been investigated only in the adult human IC (Tardif et al., 2003). Furthermore, the expression patterns of radial glial cells related to the migratory streams (Bayer and Altman, 2003), have not been investigated in the human IC.

In humans, the IC is first identifiable at 8 GW (51 days; Gardner and O’Rahilly, 1971). The cytological development of the IC has been described previously as having five major stages: 1. Neurogenesis from the posterior recess of the cerebral aqueduct (CA), 2. Migration of cells to their final positions, 3. Axons development, 4. Dendritic differentiation, and finally 5. Synapses formation (Sharma et al., 2009a). At later gestational stages, the CNIC neurons develop elongated cell bodies (“pear-shaped”) with slender processes, and where the larger neurons are twice the size of the smaller neurons (Nara et al., 1996). Afferent connections to the IC start developing around 16 GW, with commissural axons appearing around 22 GW (Moore et al., 1996). Fetal responses to auditory signals have been reported as early as 24 GW, becoming consistent after 28 GW (Birnholz and Benacerraf, 1983). Increased heart rate in response to acoustic stimulation is detected in human fetuses around 26 GW (Sharma et al., 2009a; Sharma et al., 2009b). These timelines further highlight gaps in understanding the detailed development and maturation of the IC.

Our knowledge about the neuroanatomy of the IC mainly comes from animal studies and from limited adult human specimens. The neuronal make-up of the IC is heterogenous, with at least one-fourth GABAergic neurons, which are also larger than the non-GABAergic neurons (Merchán et al., 2005; Schofield and Beebe, 2019). The CNIC, and its surrounding cortex, includes two major classes; the disk shaped and the stellate neurons. The disk shaped neurons are characterized by a flattened dendritic field, while the stellate neurons have spherical dendritic fields (Faye-Lund and Osen, 1985; Malmierca et al., 1993; Mansour et al., 2019; Meininger et al., 1986; Oliver and Huerta, 1992; Oliver and Morest, 1984). The disc-shaped neurons can be further dissociated morphologically on the basis of the thickness and density of dendritic arbors into “flat” and “less flat” neurons (Malmierca et al., 1993), they are mutually parallel, and are arranged in parallel layers in the CNIC (Geniec and Morest, 1971; Faye-Lund and Osen, 1985). The stellate neurons can be further subdivided based on the soma sizes (Geniec and Morest, 1971), and tend to be located between the CNIC layers (Faye-Lund and Osen, 1985). Furthermore, GABAergic subtypes of neurons have been identified in each of the morphological types (Ono et al., 2005). The IC nuclei, both in animals and humans, include neurons that express glutamate (Ito et al., 2015), GABA (Ito et al., 2015; Ito and Oliver, 2012; Mansour et al., 2019; Mellott et al., 2014; Ouda and Syka, 2012), CR (Imam et al., 2019; Maseko et al., 2013; Ouda and Syka, 2012; Tardif et al., 2003), CB (Imam et al., 2019; Ouda and Syka, 2012; Tardif et al., 2003), PV (Ouda and Syka, 2012; Pal et al., 2019; Sharma et al., 2009a; Tardif et al., 2003), NADPH-diaphorase (Ouda and Syka, 2012). Importantly, the calcium binding proteins, including CR, CB and PV, have been used to differentiate the classes (types) of neurons (Baimbridge et al., 1992). PV is present in metabolically active GABAergic neurons in the adult brain and is detected early in few brainstem nuclei, and tends to be expressed in the late developmental stages of other brain parts (Solbach and Celio, 1991). To the contrary, CR and CB are detected in the early stages of development (early second trimester in humans), hence they may be involved in the development of brain (Ellis et al., 1991; Enderlin et al., 1987; Hendrickson et al., 1991).

In this paper we provide a comprehensive description of the developing human IC and its parts by detailing the volumetric and cytoarchitectural development during specific stages of the second trimester: 15, 18, 21, 24, and 27 GW. We describe the maturation timeline of IC neurons using Nissl stain and CR expression patterns. We use GFAP immunohistochemistry to describe the radial glial and the astroglial development in the human fetal IC. Finally, we describe in detail the CR expression patterns in IC in each developmental stage.

## 2 Materials and methods

### 2.1 Procurement of specimens and fixation

De-identified human fetal brain specimens (deemed non-pathological) of 15, 18, 21, 24 and 27 GW, respectively, were procured from the Departments of Pathology, Mediscan Systems Pvt. Ltd. Chennai, India, and Saveetha Medical College and Hospital (SMCH) Chennai, India. Autopsies with brain extraction were performed after due consent wa obtained from the next of kin. All procedures used in this study have been performed in accordance with Declaration of Helsinki and were approved by the Institute Ethics Committee (IEC) and Institute’s Review Board (IRB) of the Indian Institute of Technology, Madras (IITM) (IEC/2021-01/MS/06) and the respective medical centers mentioned above.

The specimens were obtained within a post-mortem interval (PMI) of 2–4 h, and as a part of larger study at Sudha Gopalakrishnan Brain Center (SGBC), IITM, postmortem in-skull MRIs (1.5 and 3 T) were obtained prior to the extraction (Verma et al., 2024). The brains were extracted after 2 weeks in fixative and stored in room temperature in 4% paraformaldehyde (PFA) in 0.01 M phosphate buffer (PB) until ready for histological processing.

### 2.2 Histological processing

All the fixed whole brain specimens were cryoprotected using gradient sucrose solution, starting with 10% sucrose in 4% PFA, followed by 20 and 30% sucrose in 0.01 M PB, with a common end point of specimens attaining complete equilibrium in the solution (i.e., the brain sample submerges to the bottom of the container).

Following cryoprotection, the whole brain was frozen into customized molds previously described (Kumarasami et al., 2023), which not only enables alignment of the frozen block but also minimizes tissue distortions. Briefly, the whole brain was frozen in −80°C in an isopentane bath surrounded by a dry ice chamber. The frozen blocks were stored in −80°C refrigerator until sectioning (Pinskiy et al., 2013).

The brains were then cryosectioned using a Leica 1,520 cryostat (Leica Biosystems, India) for the smaller brains and the Leica 3600XP cryomacrotome (Leica Biosystems, India) for the larger brains. Tape transfer technique has been used for sectioning at 20 μm thickness in the sagittal and coronal planes (Pinskiy et al., 2015) and the sections were stained for three different and consecutive series, Nissl, Hematoxylin and Eosin (H&E) and Immunohistochemistry (IHC).

### 2.3 Staining and coverslipping

The first series of sections were stained in 0.2% Thionine solution (in water) (Thionine Acetate, SRL Chemicals), to label the Nissl bodies, then differentiated using distilled water, dehydrated using graded alcohol solutions and cleared with xylene prior to coverslipping using DPX (Merck, India). The second series of sections was stained with H&E using Harris’ regressive protocol. Since the fetal brains have excessive water content, all sections were dried in a temperature-controlled chamber at 33°C, prior to coverslipping.

The third series underwent immunostaining for specific antibodies with protocol specifically modified for use in the developing brain (Pandurangan et al., 2024). Briefly, the sections underwent an antigen retrieval process using boric acid buffer (pH = 9.0) at 70°C for 20 min, after which the sections were allowed to cool down at room temperature (RT). After cooling, the slides were rinsed with 0.01 M Phosphate Buffer (PB) three times for 5 min each and treated with 0.3% H_2_O_2_ solution for 20 min, to quench the endogenous peroxidase. After the peroxidase blocking, the slides were again washed three times with 0.01 M PB and a protein block was performed with animal free blocking reagent [5X Cell Signaling Technology (CST) for 1.5 h at RT. Following the protein block, the slides were incubated with IgG anti-GFAP (E4L7M) (Cat. No. 80788) XP monoclonal antibody 1:1500] or CR [Rabbit, IgG anti-CR (E7R6O) (Cat. No. 92635) XP monoclonal antibody 1:1000], for overnight and 48 h, respectively. After incubation the slides were washed 4 times with 0.01 M PB, following which the slides were incubated with Goat, anti-rabbit, IgG, polyclonal antibody (HRP conjugate, ENZO, Cat. No. ADI-SAB-300-J) at 1:500 ratio for 1.5 h at RT. Sections were rinsed thrice with 0.01 M PB and stained with a substrate containing diaminobenzidine (SignalStain DAB Substrate Kit, CST). The slides underwent dehydration with graded alcohol before being coverslipped.

For CR immunostaining in 15–24 GW, we used the DAKO EnVision secondary kit for immunohistochemistry. The sections first underwent antigen retrieval process using EnVision FLEX Target retrieval solution, High pH, for 20 min at 70°C and then cooled to RT. After cooling, the slides were rinsed in EnVision FLEX wash buffer and treated with EnVision FLEX peroxidase-blocking reagent for 10 min, followed by another rinse with the wash buffer. Subsequently, the slides were incubated for 24 h with the anti-CR primary monoclonal antibody [Rabbit, IgG anti-CR (E7R6O) (Cat. No. 92635) XP monoclonal antibody, 1:1000]. Post-incubation, the slides were washed with the buffer and incubated with EnVision FLEX/HRP for 30 min. After this step, they were washed again and stained with DAB solution (EnVision DAB + chromogen). Finally, the slides were dehydrated using graded alcohol and coverslipped.

### 2.4 Scanning and digitization

The stained sections were scanned and digitized using a large format scanner (TissueScope LE120, Huron Digital Pathology, Canada), with an in-plane resolution of 0.5 μm/pixel. These images underwent quality control tests and only those that passed the Quality Check (QC) process were considered for further analysis.

### 2.5 Identification and brain region annotation of the inferior colliculus

ICs of both hemispheres were included in our analysis. We identified the IC in 100 sections in the 15 GW specimen, 150 sections in the 18 GW and 21 GW specimens, and in over 180 sections in the 24 GW and 27 GW specimens (see Table 1). An additional sagitally cut 27 GW specimen was processed only for GFAP immunostaining. This second specimen is identified as “27 GW S2” and its results are described in Section 3.3.1.

**Table 1:** Specimen details with the number of serial Nissl-stained sections used in this study.

Age–gestation week (GW)	Plane of section	Total number of nissl sections analyzed	Brain dimensions (from postmortem in-skull MRI)
15 GW	Sagittal	100	A-P- 21.34 mm R-L- 16.57 mm
18 GW	Sagittal	150	A-P- 43.32 mm R-L- 38.93 mm
21 GW	Sagittal	153	A-P- 56.08 mm R-L-45.58 mm
22 GW	Transverse	87	A-P- 55.1 mm R-L- 42.52 mm
24 GW	Sagittal	185	A-P- 65.34 mm R-L- 57.65 mm
27 GW	Sagittal	180	A-P- 72.11 mm R-L- 59.58 mm

**Figure 1:**
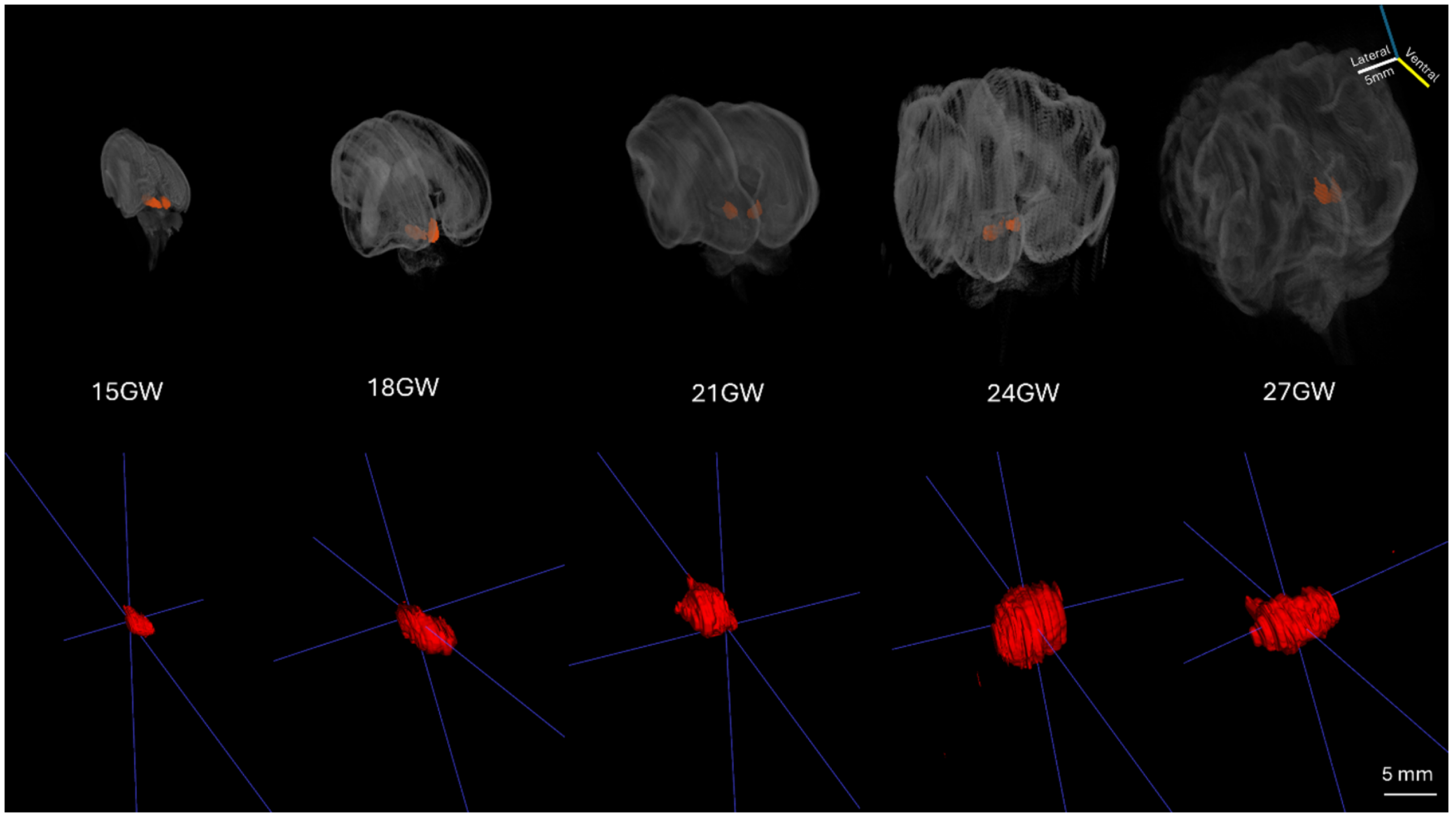
The 3D reconstructed development of the human IC during five stages of the second trimester. The top row consists of the 3D reconstructed volumes of brain specimens in all developmental stages (15 GW to 27 GW). The bottom row shows the magnified 3D volumes of the left hemisphere IC of all the brain specimens (15 GW to 27 GW).

On the basis of the previously published literature (Bayer and Altman, 2005; Bayer and Altman, 2003; Ding et al., 2022; Ding et al., 2016; Mansour et al., 2019; Olszewski and Baxter, 1982; Tardif et al., 2003), we identified and annotated IC and its subdivisions: the central nucleus (CNIC), the dorsal cortex (DCIC) and the external cortex (ECIC) along with the fiber tracts related to IC: the lateral lemniscus (LL). After the identification of the IC and based on cytoarchitectonical patterns, we annotated the different subparts of the IC, as shown in Figures 1. The adjacent H&E sections were referred to confirm the cells appearances, as well as to rule out any pathology. For comparisons reasons, we also used the marmoset atlas of Paxinos et al. (2012).

### 2.6 Stack alignment, 3D-registration and volumetric reconstruction

In order to create data volumes, the Nissl stained 2D images were first down-sampled to 60 μm per pixel, and then padded and stack aligned using the scale invariant features tool (SIFT) as a feature of ImageJ/Fiji (Lowe, 2004; RRID:SCR_002285). This aligned stack resulted in 3D volumes with 60 μm isotropic voxels, which allowed for reorientation of the brains using the following landmarks: the brain midline, the brainstem orientation, the anterior commissure (ac) and the posterior commissure (pc) line. The volumes of the whole brains (posterior view for better IC visualization) are shown in Figure 1, top row. The 3D reconstructed segmented volumes of the left ICs are displayed in the bottom row. The volumes of the left ICs (Table 2) were calculated using the segmented voxels (shown in brackets), and visualized in 3D viewers such as MATLAB (see Figure 1).

**Table 2:** Volumes of the left IC of all the specimens from 15 GW to 27 GW in mm^3^, as well as voxels.

Gestational age (GW)	Left IC Volume (in mm^3^) Voxel size = 60 μm x 60 μm x 60 μm
15GW	12.85 (59,484 voxels)
18GW	18.01 (83,372 voxels)
21GW	19.61 (90,772 voxels)
24GW	33.21 (154,268 voxels)
27GW	34.27 (158,673 voxels)

## 3 Results

### 3.1 The morphological development of the IC

The 3D reconstruction of histological sections was used to evaluate the increase of the IC in the five considered stages in the second trimester. Figure 1 shows the whole brains 3D volumes with the IC highlighted, while the bottom row displays the left IC of all brain specimens ranging from 15 GW (extreme left) to 27 GW (extreme right). The overall IC volume increases approximately threefold from 15 GW (12.85 mm^3^) to 27 GW (34.27 mm^3^).

From 15 GW to 18 GW, the IC volume increases sharply by about 1.5 times, whereas there is a minimal rise of only 1.60 mm^3^ from 18 GW to 21 GW. A large increase of almost 60% is also observed between 21 GW and 24 GW, when the IC volume grows from 19.61 mm^3^ to 33.21 mm^3^, respectively. Finally, there is a small increase of only 1.06 mm^3^ from 24 GW to 27 GW.

As described above, the volumetric increase of the developing human IC is not linear, but occurs in two major stages; the first during the 15–18 GW period, and the second in the 21–24 GW period.

**Figure 2:**
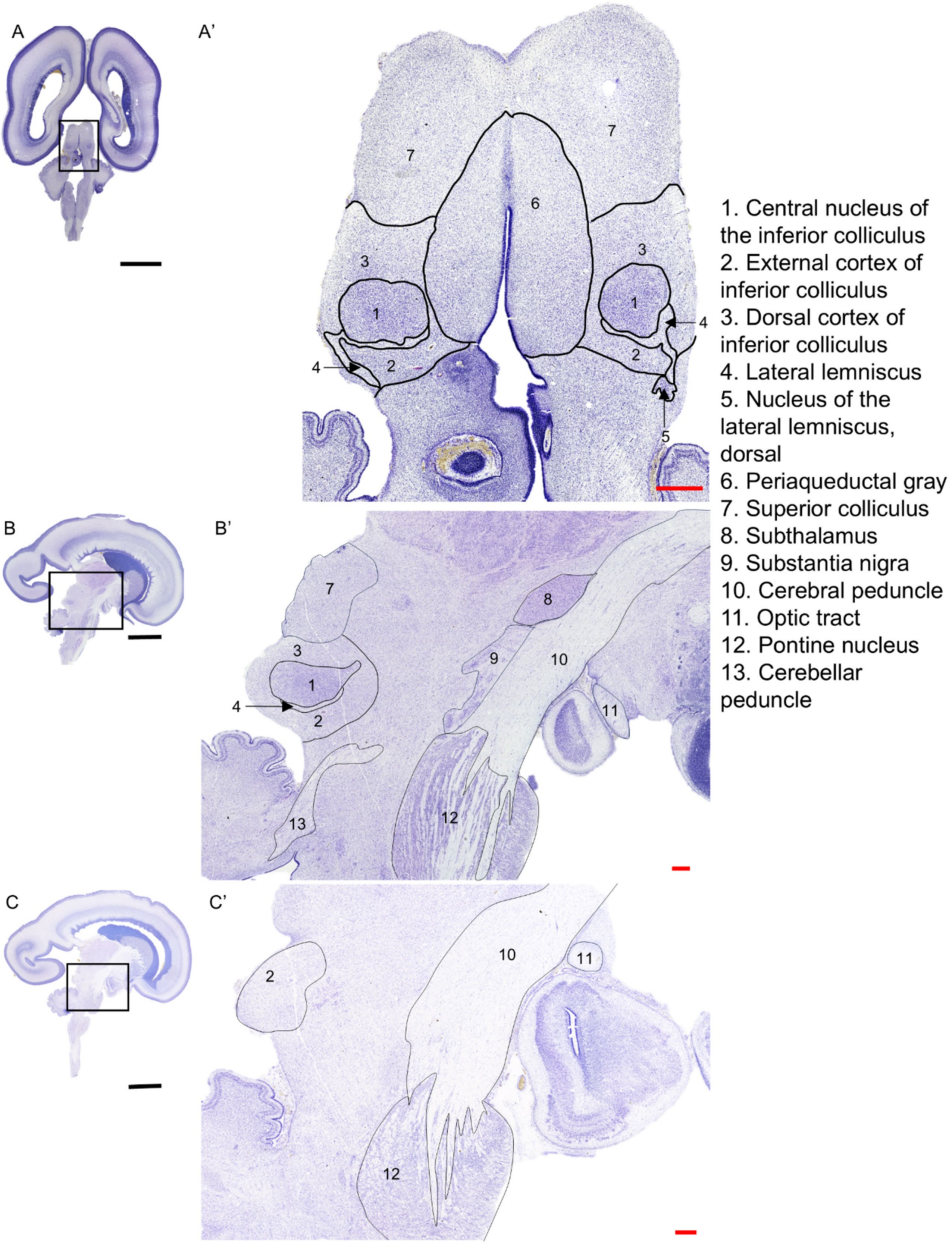
Nissl-stained sections of the 22 GW brain in transverse plane **(A)**, and 21 GW brain in sagittal plane **(B,C)** that include the IC nuclei: CNIC, DCIC, ECIC, as well as the neighboring brain regions and fiber tracts. The black scale bar indicates 1 cm, and the red scale bar indicates 1 mm.

In Figure 2, the IC, its subparts, and the neighboring structures are shown both in sagittal (21 GW) and transverse (22 GW) sections. The CNIC appears as a densely packed structure with a roughly circular shape in the transverse section (Figure 2A), lateral to the periaqueductal gray (PAG). In sagittal view, it appears as an almond-shaped structure (Figure 2B), demarcated rostrally (anteriorly) by the superior colliculus (SC; Figure 2B). The DCIC is identified in both sagittal and transverse planes, superior (dorsal) to CNIC [Figures 2A,B; (Geniec and Morest, 1971; Tardif et al., 2003)]. A portion of the ECIC is present in the caudal (posterior) side of the CNIC separated by a thin part of LL. ECIC is lined by a prominent white matter tract laterally (LL), and by the nucleus of lateral lemniscus, dorsal (NLLd) in its inferior part (ventrally; Figure 2A). The ECIC is present ventrally to CNIC in the sagittal plane and it extends further laterally (about 1.1 mm from the midline; see Figure 2C).

**Figure 3:**
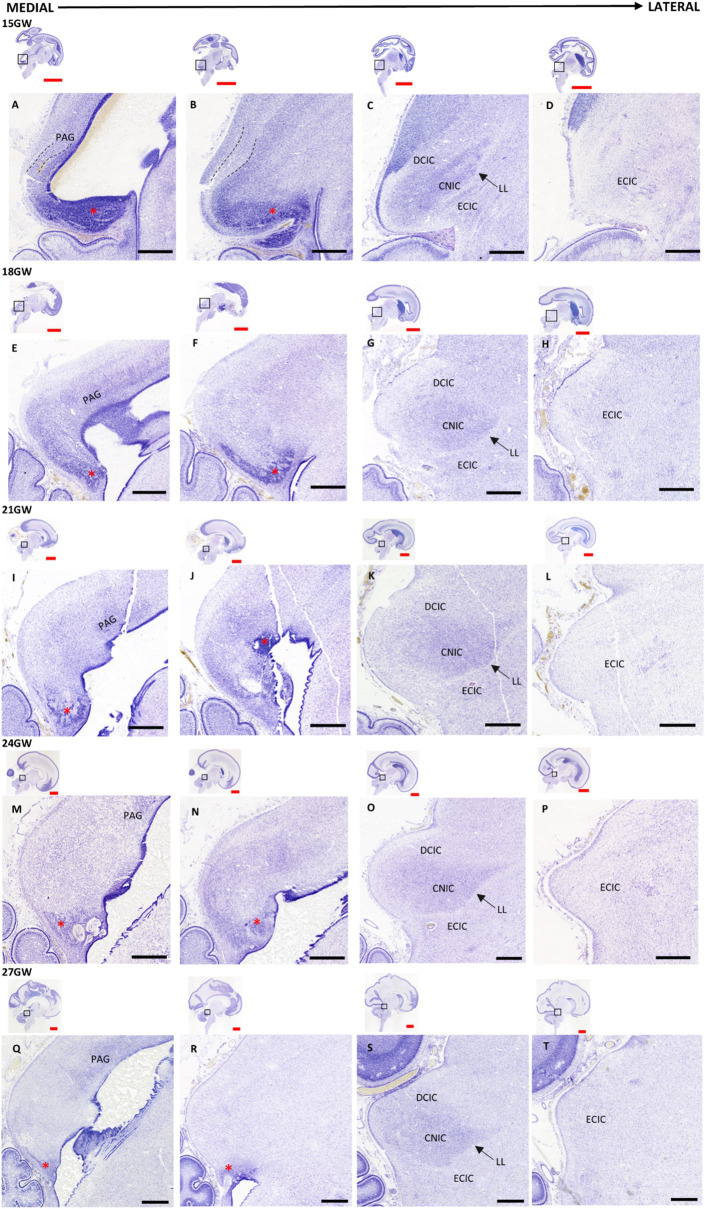
Nissl-stained sagittal sections of the human fetal brain 15-27 GW. The IC and its neighboring regions, and LL displayed medially to laterally at different gestational ages: 15 GW **(A–D)**, 18 GW **(E–H)**, 21 GW **(I–L)**, 24 GW **(M–P)**, and 27 GW **(Q–T)**. Each column represents the approximate same medio-lateral position, as shown in the sagittal insets. The mesencephalic neuroepithelium **(M,N)** (red asterisk), located in the posterio-inferior part of the CA is prominent at 15 GW **(A)**. It gradually decreases in the subsequent developmental stages **(E,I,M,Q)**. The continuation of the MN can be seen in the inferior part of IC, which is markedly present in 15 GW **(B)**, it is gradually reduced in size at 18, 21 and 24 GW **(F,J,N)**, respectively, and it disappears at 27GW **(R)**. The central nucleus **(C,G,K,O,S)** occupies most of the IC and appears as a rough oval shape structure with a clear ventromedial boundary lined by a layer of LL (marked by black arrows), and with a more diffuse dorsolateral boundary. See text for details (3.1). Black scale bar indicates 1mm. Red scale bar indicates 1cm.

At 15 GW, the MN is very thick and covers a large portion of the ventral IC. Two layers of cells arise from the MN, an outer and an inner layer, and are separated by a cell sparse layer (see Figures 3A,B). The inner layer of cells converges about 1.4 mm from the CA laterally, into the oval shape forming the CNIC (see Figure 3C). The MN gradually decreases in the older developmental stages and becomes almost negligible in 27 GW. We also observe a small PAG posterior to MN at 15 GW (Figure 3A), which increases in cytoarchitectural density at 18 GW, and gradually decreases in 21 (Figure 3I), 24 (Figure 3M) and 27 GW (Figure 3Q).

CNIC is the most prominent part in the adult IC, and it can be detected in the 15 GW specimen as an oval shape with a distinct dense cellular layer relative to the overall appearance of the nucleus (Figure 3C). A streak of thin white matter, identified as the LL, marks the ventromedial boundary of the CNIC whereas there is a diffuse dorsolateral boundary with the DCIC (Figure 3C). The shape of CNIC becomes more prominent with GW (Figures 3C,G,K,O,S). The cytoarchitectural boundaries of DCIC cannot be clearly demarcated at 15 GW. However, DCIC can be distinguished as a distinct group of cells capping the superior (dorsal) part of the CNIC at 18 GW (Figure 3G). It develops morphologically and cellularly at 21 GW (Figure 3K), at 24 (Figure 3O), and at 27 GW (Figure 3S). As the DCIC develops, the boundary of CNIC with DCIC becomes more distinct with increasing GW. ECIC can be seen as a group of cells inferior to CNIC, separated by LL, which also continues further laterally (Figures 3D,H,L,P,T). There are no visible cytoarchitectural patterns in ECIC at 15 GW, apart from a few cellular aggregations which may be migrating cells. In the subsequent stages, ECIC does not undergo any major changes cytoarchitecturally, and appears as a uniform cellular field that is sparse when compared with CNIC.

At 24 and 27 GW, we see a cell-sparse layer which separates IC from SC (the intercollicular area; Figures 3O,S; Nara et al., 1996).

### 3.2 Stages of cellular maturation

**Figure 4:**
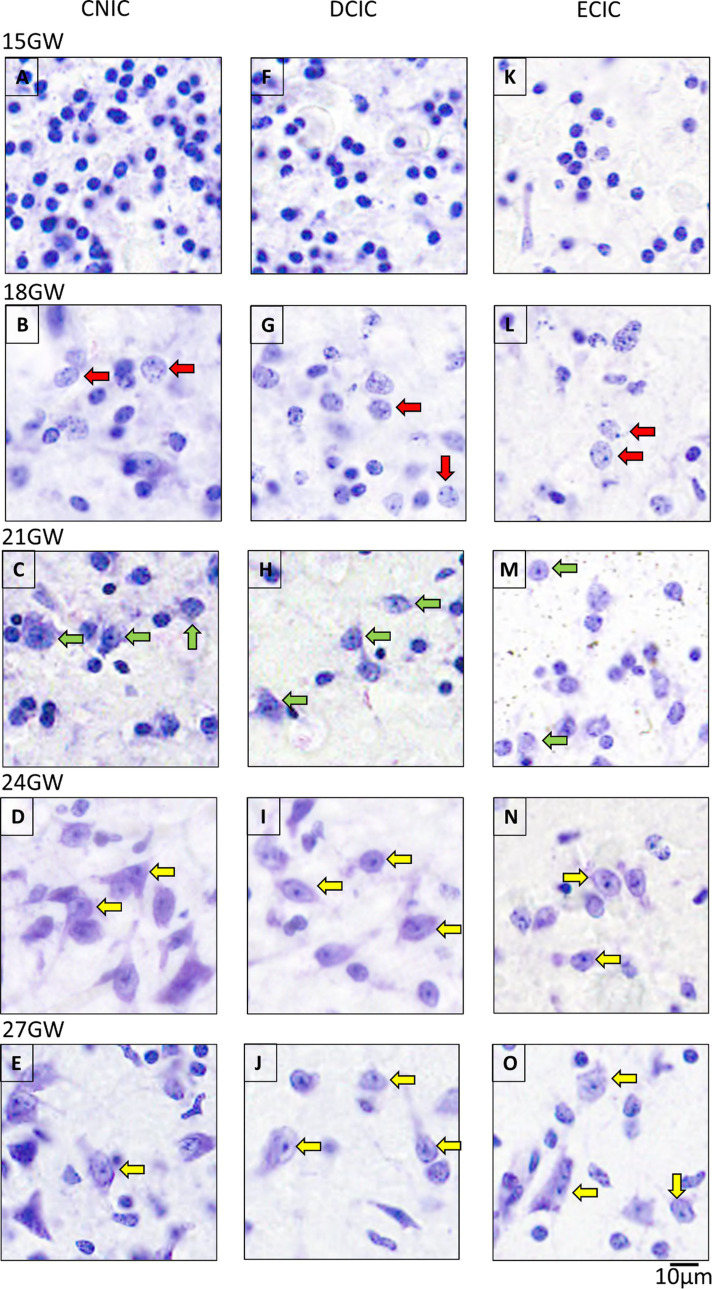
The maturation pattern of neurons in CNIC, DCIC and ECIC. At 15 GW, IC has immature cells characterized by spherical and uniformly stained somas **(A,F,K)**. At 18 GW **(B,G,L)**, some cells have a spotted appearance inside the somas (red arrows). At 21 GW, the neurons start to show distinct nucleoli [**(C,H,M)**; green arrows]. By 24 GW **(D,I,N)**, and 27 GW **(E,J,O)**, mature neurons have elongated or spherical cell bodies, and with prominent single nucleoli in the middle (yellow arrows). The scale bar is 10 μm.

Serial and high-resolution images of Nissl preparations (see Table 1) were used to study the maturation of cells in all three IC nuclei. The various stages of neuronal differentiation (Šimić et al., 2022) are described below:

At 15 GW, the IC includes only undifferentiated and uniformly stained cells (Figures 4A,F,K). At this stage, the neurons cannot be differentiated from the glia. At 18 GW, few cells have circular somata larger as compared with 15 GW (Figures 4B,G,L), and scattered in all IC nuclei. At 21 GW (Figures 4C,H,M), the neurons start to show a single nucleolus in the middle of the cell body, which distinguishes them from other classes of cells. At 24 GW (Figures 4D,I,N) and 27 GW (Figures 4E,J,O), neurons appear as flat enlarged cells (compared to the surrounding glia), with a prominent and dense single nucleolus and a sparsely stained nucleus (see Nara et al., 1996).

Qualitatively, the overall density and number of cells appeared highest in CNIC, followed by DCIC, and the lowest in ECIC.

**Figure 5:**
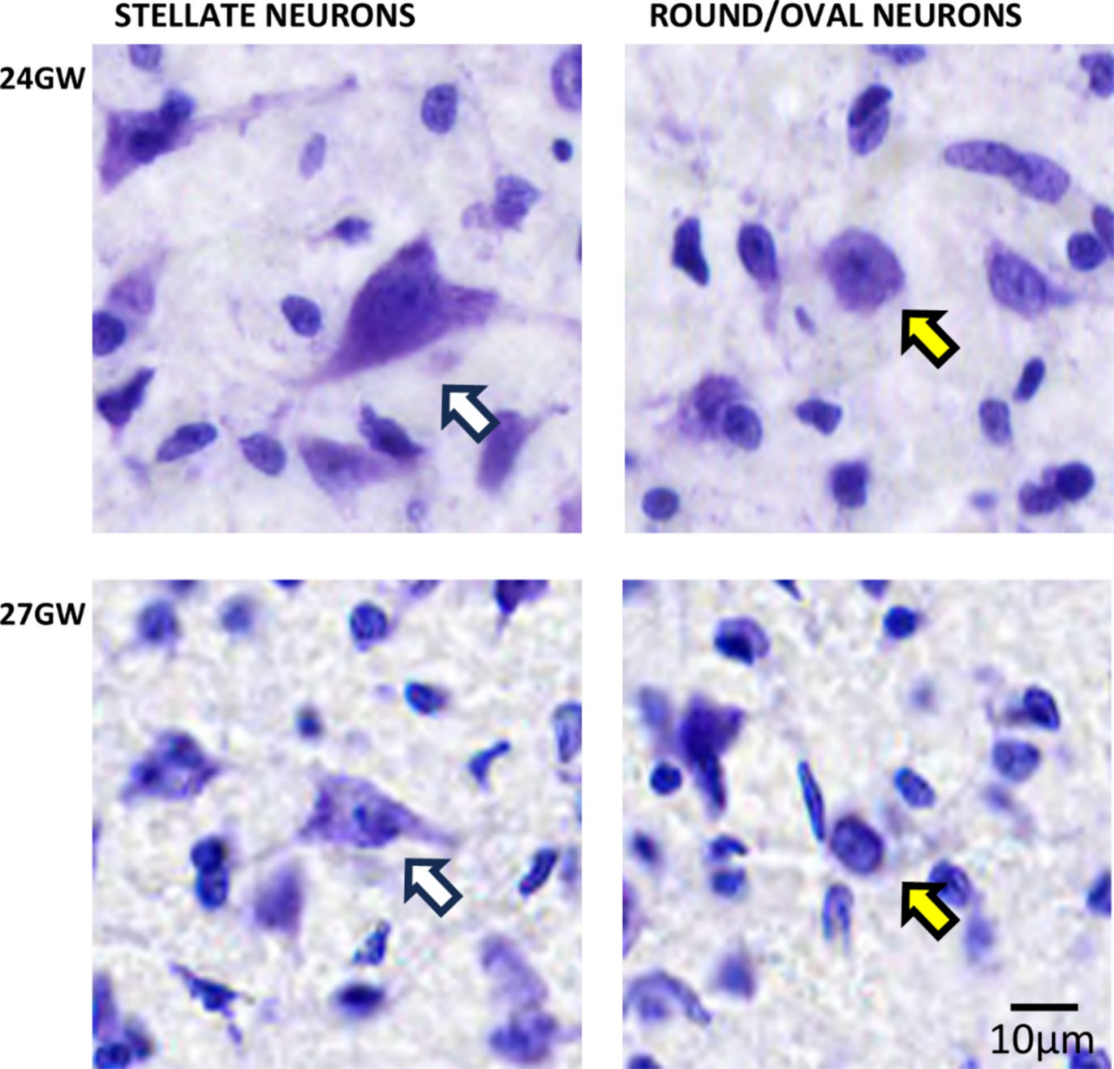
Triangular and polygonal shaped (stellate-like; white arrows) and round/oval (yellow arrows) neurons in CNIC identified at 24 and 27 GW, respectively. See text for details. The scale bar is 10 μm.

In the older specimens of 24 GW and 27 GW, based on the cell body shape, we identified two distinct neuronal populations: triangular or polygonal looking shape (stellate-like) and round/oval with circular looking cell body shapes (see Figure 5). These neuronal populations may correspond to those described in the adult human (Mansour et al., 2019), and the rat CNIC (Malmierca et al., 1993). Nevertheless, further experiments and individual cells reconstructions are needed to better identify these neurons (see Malmierca et al., 1993). Stellate-like neurons have been primarily identified in CNIC, and round/oval neurons in DCIC and ECIC.

### 3.3 Immunohistochemistry

#### 3.3.1 GFAP immunohistochemistry

**Figure 6:**
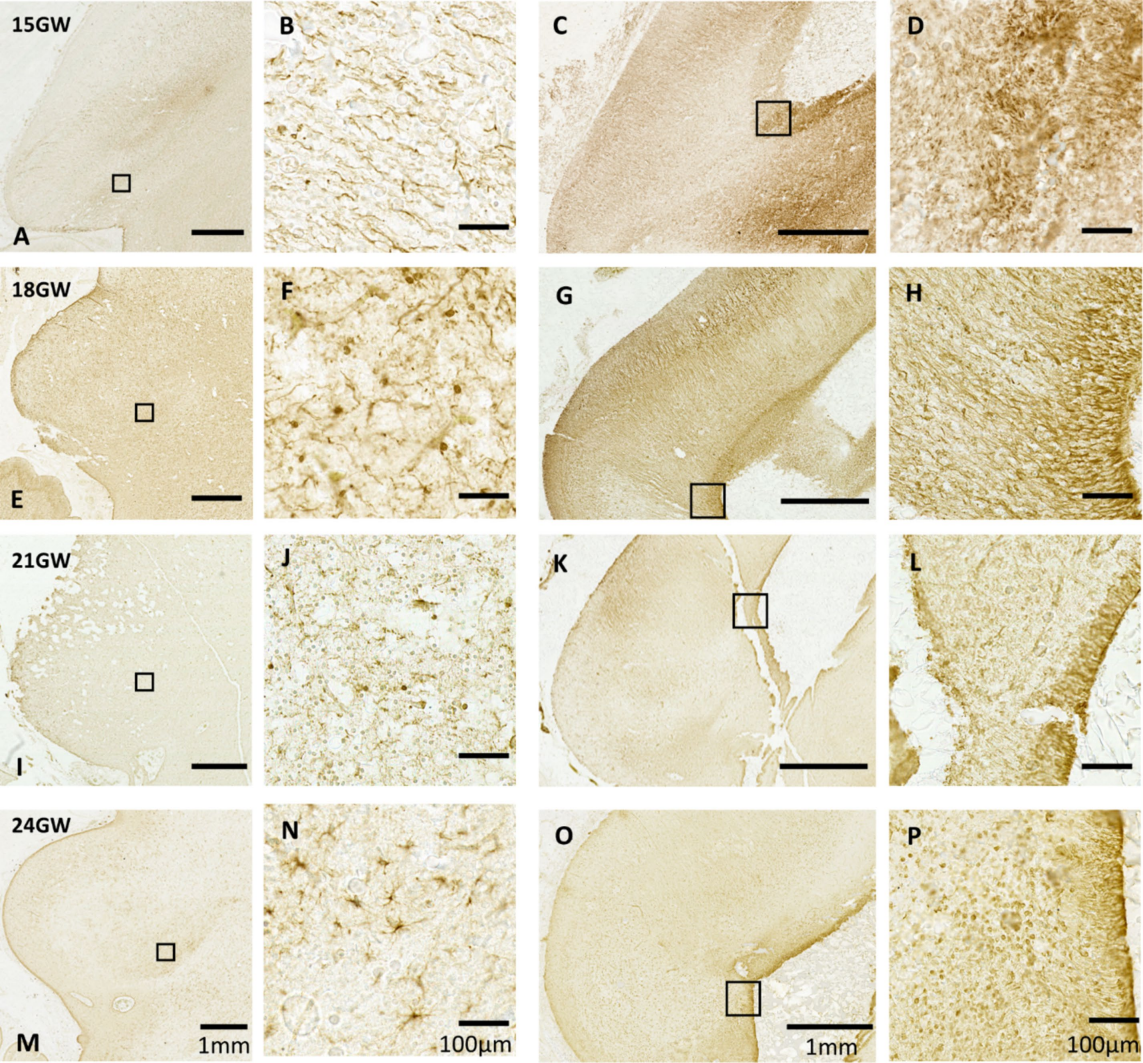
Anti-GFAP immunohistochemistry in the human fetal tectum. Each of the rows represents a specific age group. The left two columns **(A,B,E,F,I,J,M,N)** display GFAP expression in IC, and the right two columns **(C,D,G,H,K,L,O,P)** show GFAP expression patterns near CA in the mid-sagittal sections. **A–D** (15GW): only radial glial fibers have been identified. With dense and long radial fibers in the mid sagittal section **(C,D)**. **E,F** (18 GW), **I,J** (21 GW): few astroglia could be identified. **M–P** (24GW): identifiable cell bodies with distinct processes that are identified. Tapered radial glial fibers are present in **L** (21 GW) and **P** (24 GW), respectively.

In order to investigate the development of astrocytes and the spread of radial glia in cell migration, we examined the distribution of the glial fibrillary acidic protein (GFAP) in the IC and its subparts. GFAP is present in radial glial fibers that play a crucial role in the migration and specification of cells during brain development (Casper and McCarthy, 2006; Kostović et al., 2018; Kriegstein and Götz, 2003; Rakic, 1972). Starting with the 15 GW specimen, GFAP immunoreactivity did not delineate any boundary between the CNIC, DCIC and ECIC (see Figure 6A). We identified bands of radial glial fibers, but no signatures of astroglia in the IC (Figure 6B). Furthermore, we could identify the origin of these radial glial fibers in the mid-sagittal region of tectum of the same specimen in the vicinity of the CA (Figure 6C). This pattern remains consistent at 18 GW in IC, without any possible distinction of subparts of the IC (Figure 6E); however, scattered astroglia were found (Figure 6F).

**Figure 7:**
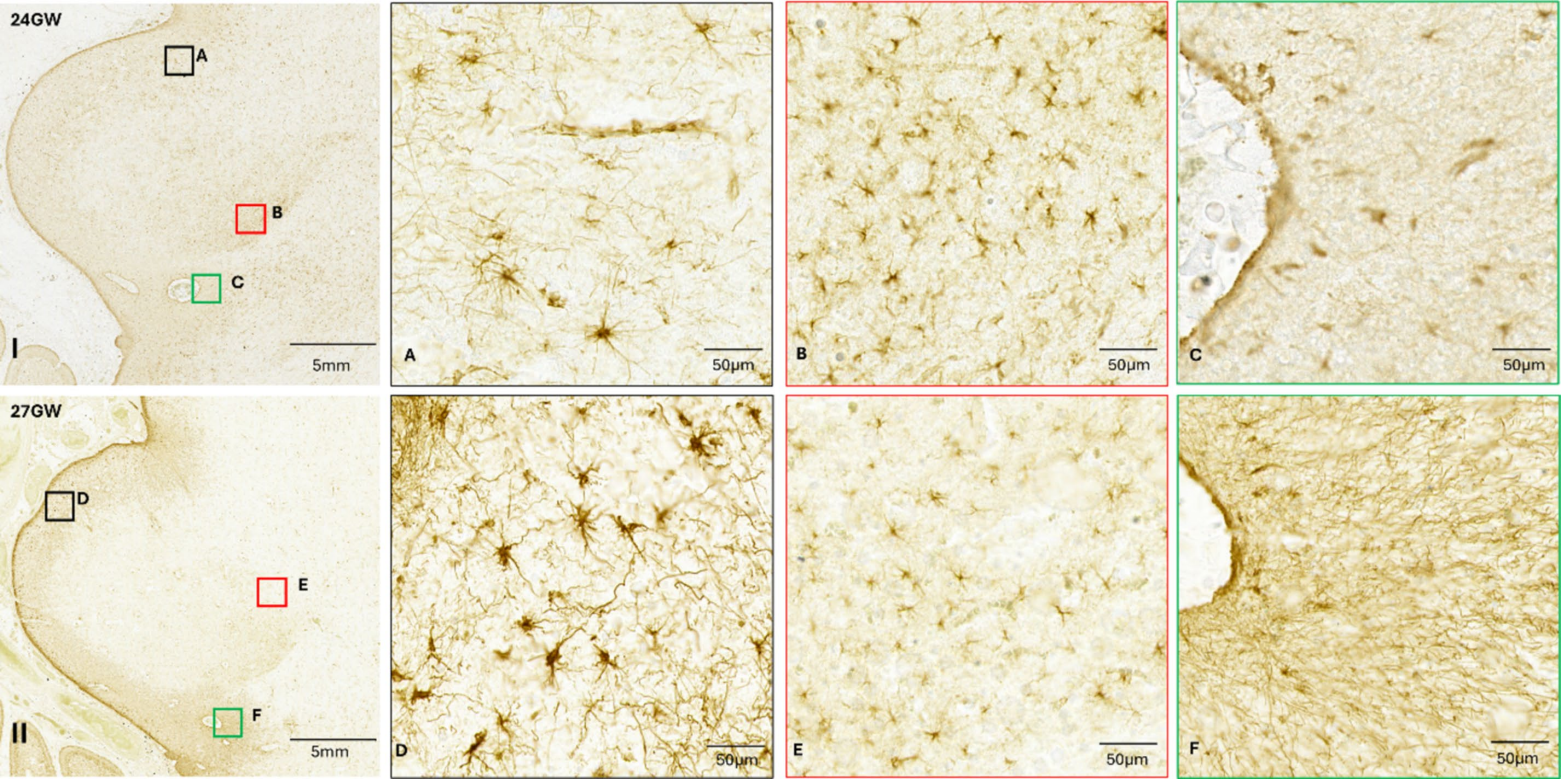
Anti-GFAP immunohistochemistry performed in the IC of the 24 GW and 27 GW (S2) specimens. I and II: low magnification images of the IC. **(A,B)** insets taken of 24 GW IC. **(D,E)** insets of 27 GW (S2) IC. Green squares indicate astroglial fiber networks around blood vessels **(C,F)**. See text for details.

At 21 GW (Figure 6), the identified astroglia are distributed in a similar fashion as in 18 GW (Figure 6J). Mid-sagitally, the radial glial fibers are tapered off resulting in the GFAP staining becoming lighter (Figures 6K,L). The mid-sagittal region of IC at 24 GW (Figures 6O,P) shows a decreasing pattern in the radial glial fibers as compared to 21 GW, but we also see a dense distribution of astrocytes close to the CA. Unlike 21 GW (Figure 6I), when IC has a uniform distribution of GFAP, at 24 GW (Figure 7I) and 27 GW (Figure 7, II) the CNIC starts to differentiate from the rest of the cortex. Furthermore, an accumulation of astrocytes and glial fibers was visible over the infero-medial boundary of the CNIC at 24 GW (Figure 7, IB) which continued at 27 GW (Figure 7, II D). We also observed a group of scattered astrocytes along with glial fiber network in DCIC in 24 GW (Figure 7A). This layer becomes much denser in 27 GW (Figure 7C). These glial fibers networks form around major blood vessels at 24 GW (Figure 7I, green squares), which becomes more prominent at 27 GW (Figure 7, II, green squares).

#### 3.3.2 CR immunohistochemistry

**Figure 8:**
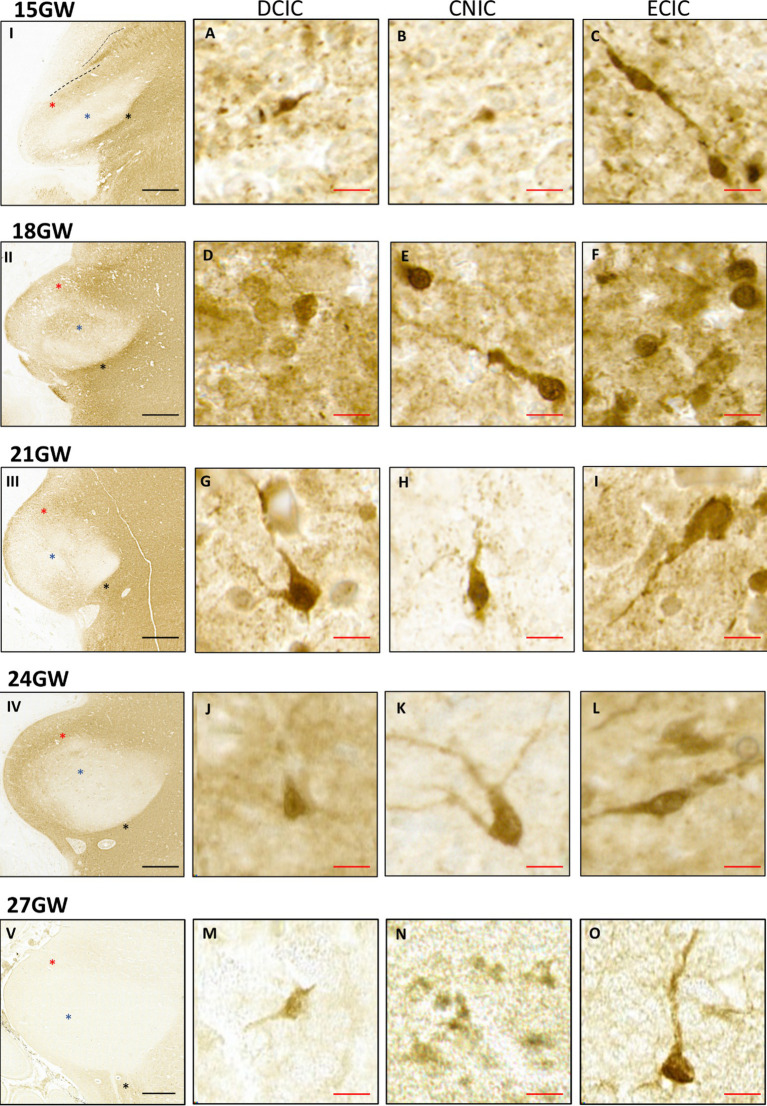
Anti-CR immunohistochemistry in the developing human IC, from 15 GW to 27 GW (I - V). Red asterisks indicate DCIC, blue asterisks indicate CNIC and black asterisks indicate ECIC. The CNIC includes only few scattered CR positive neurons. However, the surrounding cortex has neuropil labeling. The DCIC has less intense neuropil staining as compared to ECIC. The number of CR positive neurons in the dorsal cortex is highest at 15 GW **(A)** and gradually decreases at 18 GW **(D)**, 21 GW **(G)**, 24 GW **(J)**, and at 27 GW **(M)**, respectively. Most of the CR-positive neurons in DCIC are small, and the staining is present only in the soma. Comparatively, the ECIC has a denser neuropil staining and more CR positive neurons stained along with their processes **(C,F,I,L,O)**. Black scale bar indicates 1 mm. CNIC is mostly devoid of neuropil staining. It shows a few CR positive neurons in 15 GW **(B)**, 18 GW **(E)**, 21 GW **(H)**, 24 GW **(K)** and no positive neurons in 27 GW **(N)**. Red scale bar indicates 10 μm.

CR plays an important role in the development and functioning of neurons in the central nervous system (CNS). It has been used to study the development and maturation of neurons in different parts of the human fetal brain, and in the mammalian IC (Baimbridge et al., 1992; Lohmann and Friauf, 1996; Schwaller, 2014), but not in the developing human IC. Hence, we have conducted anti-CR IR in the CNIC, DCIC, ECIC, to study the maturation of neurons in different developmental stages (see Figure 8).

At 15 GW (Figures 8A–C, I), there is no CR+ neuropil staining in the CNIC, and the CR+ neurons are scattered only near its border with DCIC (Figure 8B). The labeled neurons have a roughly round shape. In DCIC we observed neuropil staining in the entire nucleus, which divides it in two specific parallel bands, as shown in Figure 8, I. We also observed several small scattered cells across the entire nucleus (Figure 8A). In ECIC, posterior to CNIC, we observed dark neuropil staining with high density of neuronal cell bodies along with scattered neuronal processes (Figure 8C).

At 18 GW (Figures 8, D–F, II), the CNIC continues to have negligible neuropil staining except in the central region. CR+ neurons are scattered across the entire nucleus (Figure 8E). Furthermore, the neuropil staining covers the inferior part of the CNIC, with neurons scattered randomly (Figure 8F). Most of these neurons have a lighter core with a darker boundary surrounding them, and few of them are stained uniformly dark and with CR-labeled processes (Figures 8E,F). DCIC has a darker and uniform neuropil staining with scattered CR+ neurons, as compared to the 15 GW specimen (Figure 8D). ECIC also has a dense neuropil staining and scattered CR+ neurons (Figure 8F).

At 21 GW (Figures 8G–I, III), the CNIC continues to have negligible neuronal stain except for a small patch at the inferior end. The labeled neurons are randomly scattered throughout the CNIC (Figure 8H). The neuropil stain decreases in DCIC, as compared to the younger age group. The CR+ neurons are fewer as compared to 18 GW, and they are evenly distributed throughout the nucleus (Figure 8G). In ECIC there is a dense neuropil staining (Figure 8I) with several scattered neurons. By this developmental stage all observed CR+ neurons have been strongly and uniformly labeled, both the soma as well as the processes (Figures 8G–I).

At 24 GW (Figures 8J–L, IV), the CNIC includes only few labeled and randomly scattered neurons (Figure 8K). In the DCIC the neuropil staining is similar as compared to 21 GW (Figure 8J). In ECIC there is very uniform neuropil staining with several randomly scattered CR+ neurons, with both unipolar and multipolar appearances (Figure 8L).

**Figure 9:**
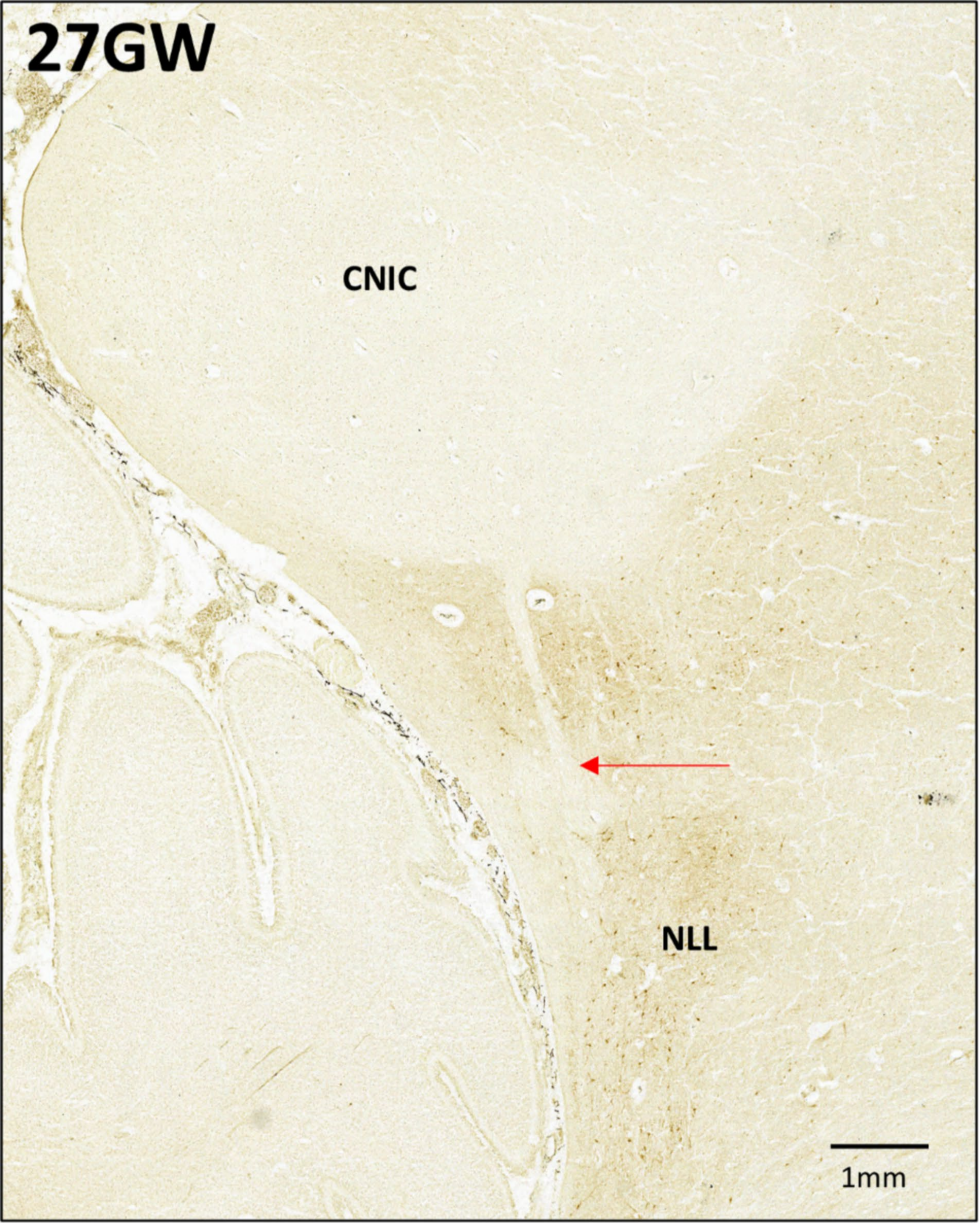
Anti-CR immunohistochemistry in the 27GW specimen. Putative LL fibers from the NLL are seen entering the CNIC (marked by red arrow).

At 27 GW (Figures 8M–O, V), the CNIC includes few labeled neurons (Figure 8M). The DCIC has very light neuropil staining, and because of this the boundary between DCIC and CNIC becomes very difficult to determine. Few CR+ neurons are found scattered in the DCIC (Figure 8M). ECIC has a light neuropil staining with scattered CR+ neurons. (Figure 8O). Moreover, CR negative LL fibers have been observed entering the CNIC only at 27 GW (See Figure 9; black arrow).

## 4 Discussion

In this paper we provide a detailed description of the human fetal IC across 5 stages of the second trimester of development: from 15 GW to 27 GW. Using Nissl-stained high resolution serial sections, we describe the shape and gross morphology of the developing IC and its subparts. For each stage, we have manually analyzed between 100 to 180 serial Nissl-stained sections, which also allowed us to track in detail the cytoarchitectonical development of the human IC. Furthermore, this is the first investigation of voxel-based 3D reconstructed histological sections, and of GFAP and CR immunohistochemistry in the developing human IC, to our knowledge. We have evaluated cellular maturation in each IC subparts using Nissl and CR IR.

### 4.1 The morphological development of IC

The literature about the neuroanatomical features of adult human IC provides information about different parts of the IC, and their neuronal architecture (Geniec and Morest, 1971; Mansour et al., 2019). We have used the existing literature of adult human IC to identify and compare the development of IC and its subparts in all our histological sections of all specimens. At a gross anatomical level, CNIC occupies the major part of the IC and a thin layer of cortex surrounds it. The CNIC appears as a dense ovoid patch of cells differentiating it from ECIC and PAG by the developing LL, as described earlier in adult IC (Mansour et al., 2019). The ovoid shape is clearly distinguishable from 21 GW, but the dense cellular organization of the CNIC and the developing LL can be identified in our youngest specimen of 15 GW.

In order to understand the spatial dynamic of the developing IC, we performed volumetric measurements, using isotropic voxels of 60 μm on aligned histological sections. This provided a precise measurement of the IC volume. We observed continuous growth of the developing IC from 15 GW to 27 GW, with the left hemisphere IC at 27 GW measuring 34.27 mm^3^, nearly three times its volume at 15 GW (12.85 mm^3^). This finding contrasts with previous literature (Nara et al., 1996), which reports an IC volume of approximately 5.5 mm^3^ at 27 GW. Several factors may account for this discrepancy. First, Nara et al. may have measured the CNIC volume only, and we included in our measurements all three IC nuclei. Second, our use of isotropic voxels of 60 μm accounts for the unique shape and size of the IC, providing a more accurate volume estimate compared to the Nara et al. method of calculating volume by multiplying the mean area by the nuclei length and considering it as a column. Last, we used cryosections for Nissl staining and volume calculation, with negligible shrinkage, whereas Nara et al. used celloidin blocks, which can shrink the tissue non uniformly by 10% (Nara et al., 1996).

The literature about the development of IC reports accelerated growth of IC from 18 GW (Nara et al., 1996; Sharma et al., 2009a). In the 18 GW specimen, we see an almost two-fold increase in the thickness of the tectum in the sagittal view, with a dense arrangement of radially arranged cells around the CA. We hypothesized this may be at least partially explained by an increased migration pattern around this age. Therefore, we have employed GFAP-IR, which is commonly used to study the developing brain (Rezaie et al., 2003; Sharma et al., 2009b; Verma et al., 2024). GFAP+ radial glial fibers originate from the ventricular zone from 15 GW onwards in a general pattern that resembles that of the developing cerebral cortex. However, the radial glial fibers density in the tectum peaks at 18 GW, and then it is gradually reduced. The development shown by GFAP labeling was confirmed by CR immunohistochemistry. The CR IR at 18 GW reveals a high amount of neuropil staining in all parts of the IC cortex, including the middle portions of CNIC, and compared to other developmental stages. Moreover, CR-positive neurons are spread randomly across the CNIC. This implies that the expression of CR is high at this stage, and it may play an important role in the migration of the neurons.

Nevertheless, the hypothesis discussed here needs further confirmation from more stages during the second trimester of development, as well as within each stage, in such way that statistical analysis becomes possible.

### 4.2 Neuronal differentiation of developing human IC

We have imaged all sections at 0.5 μm/pixel resolution (see Table 1), offering a detailed view of intracellular staining, which allowed differentiation between various developmental stages. Overall, the neuronal differentiation in the IC follows a similar trajectory as in other parts of the brain (Šimić et al., 2022). By 18 GW they reach an intermediate stage with multiple Nissl-stained nucleoli, resembling developing astrocytes. Finally, by 24 GW, the IC neuron bodies begin to take polygonal or round/oval shapes (Geniec and Morest, 1971; Mansour et al., 2019). Different from the adult (Mansour et al., 2019), we identified neurons with polygonal cell bodies only in CNIC, but not in the cortical part of the nucleus. One limitation of this study is that of the employed methods: Nissl staining is not the most suitable one to study detailed morphology of neurons. Other types of staining, such as Golgi staining, as well as quantitative analysis, are required to properly classify and describe these neurons. Moreover, it is possible that stellate neurons develop in different parts of the IC at different time points of development, which might depend on other factors, such as the development of white matter fiber tracts (LL; see also Figure 9).

### 4.3 Astroglial differentiation of developing human IC

GFAP-IR patterns described above provide information about the development and differentiation of astroglia in the 15–27 GW human fetal IC. Contrasting to the radial glial trajectory in the developing IC, we observed an increase of the astroglia development by the gestational age. The GFAP immunoreactive astrocytes are first observed at 18 GW. This developmental stage may be a point of transition when the glial cells start losing their fibers and begin to differentiate into astrocytes. This may be an initial phase of differentiation, as the astrocytes identified at this stage are spherical, and do not have any processes. As described above, the astrocytes become abundant by 24 GW and this continues at the 27 GW. In these developmental stages, the astrocytes appears to be more mature as they have smaller cell bodies and long processes, giving them a typical look as reported earlier in different parts in the developing brain, and in the adult brain (Kostović et al., 2018; Rezaie et al., 2003). This is supported by *in situ* data, which suggest that in the IC the astroglia start to differentiate after 16 GW (Sharma et al., 2009b). During 24 GW and 27 GW, the astrocytes tend to cluster around the ventromedial boundary of CNIC lining the LL, which may indicate the start of innervation of CNIC by LL. This was also observed using CR immunoreactivity in the 27 GW specimen (see also Figure 9). However, further evidence is needed to confirm it. Nevertheless, this is the first study to our knowledge that details the dynamics of both radial glia and astrocytes in the second-trimester human fetal IC.

### 4.4 CR expression patterns in the developing human IC

Calcium-binding protein expression patterns are involved in neuronal differentiation, and they have been previously investigated in the IC (Tardif et al., 2003). We have CB and PV immuno-stained sections of the 22 GW coronally cut specimen, and the results are similar with those already reported in the literature (Sharma et al., 2009a). Hence, we report here only the CR expression patterns in the human fetal IC during the second trimester of development. As described above, there are several common and specific features in the general pattern of CR IR across all five developmental stages. The major common feature is the pattern of neuropil staining. In all developmental stages, the CNIC has negligible neuropil staining unlike the cortex. This particular pattern of CR staining has been reported in the adult IC (Tardif et al., 2003). Furthermore, at 27 GW, an intense staining in the NLLd was observed.

Finally, different patterns of neuronal staining are present in different parts of the IC nuclei, and at different gestational ages, which may indicate specific stages of neuron maturation. The spatial specificity of CR+ neurons may also suggest that topographically distinct neuron types or classes may develop and mature at different speeds in the human IC.

To summarize, the development of the human fetal IC during the second trimester can be described as occurring in two major stages. During the first stage, between 15 GW and 18 GW, the process of migration is prevalent, as well as the density of GFAP+ radial fibers. At this stage the cells are immature, and the growth of the IC may be attributed to the high density of radial glial fibers. The second stage of growth occurs between 21 GW and 24 GW, when the process of maturation of cells becomes prevalent, especially in the CNIC, and the morphological change of the IC may be attributed to this. During this stage, the migration process tapers down, and the immature cells start to develop into neurons of different classes, and into glial cells. Overall, the second trimester is therefore a crucial period for the neurotypical development of the human IC.

## Data Availability

The original contributions presented in the study are included in the article, further inquiries and data requests can be directed to the corresponding author upon reasonable request.
